# Virtual Reality for the Improvement of Perioperative Care in Otorhinolaryngology-Related Procedures: A Systematic Review and Meta-Analysis

**DOI:** 10.7759/cureus.84238

**Published:** 2025-05-16

**Authors:** Ali Alseneid, Marwan Ibrahim, Alhassen Alseneid, Punsiri Silva

**Affiliations:** 1 Ear, Nose and Throat (ENT), University of Buckingham Medical School at Milton Keynes University Hospital, Milton Keynes, GBR; 2 Ear, Nose and Throat (ENT) Head, Neck and Thyroid Surgeon, Milton Keynes University Hospital, Milton Keynes, GBR

**Keywords:** head and neck surgery, immersive technologies, otorhinolaryngology, pain management, perioperative care, systematic review and meta-analysis, virtual reality

## Abstract

Virtual reality (VR) has emerged as a promising non-pharmacological adjunct for perioperative care, offering potential benefits in alleviating symptoms such as postoperative pain and reducing anxiety. However, its use in otorhinolaryngology (Ear, Nose and Throat (ENT)) surgical settings remains insufficiently explored. This systematic review and meta-analysis aimed to evaluate the efficacy of VR interventions compared to standard care across key perioperative outcomes in ENT and head and neck surgery.

This review adhered to the Preferred Reporting Items for Systematic reviews and Meta-Analyses (PRISMA) 2020 guidelines and was registered in PROSPERO. Comprehensive searches were conducted in PubMed, Embase, and Cochrane Library from inception to April 2025. Studies were eligible if they compared VR interventions to control groups in ENT perioperative settings. Risk of bias was assessed using RoB 2 and ROBINS-I tools, and data synthesis was performed using a random-effects meta-analysis.

A total of nine studies involving 527 participants were included. The postoperative pain scores demonstrated a statistically significant modest reduction, favoring VR (standardized mean difference (SMD) = -0.41; 95% confidence interval (CI) -0.78 to -0.05), with low heterogeneity (I² = 16.2%). A subgroup analysis for postoperative pain scores indicated a larger effect in pediatric populations (SMD = -0.59; 95% CI -3.70 to 2.51) compared to adults (SMD = -0.38; 95% CI -0.99 to 0.23), although neither subgroup reached statistical significance. A sensitivity analysis was also conducted for the postoperative pain scores which strengthened the findings (SMD = -0.47; 95% CI -0.80 to -0.13), and showed no observed heterogeneity (I² = 0%).

For perioperative anxiety, VR interventions offered a slight reduction compared to standard care (SMD = -0.85; 95% CI -1.88 to 0.18), though this did not reach statistical significance. Patient satisfaction scores showed a small but statistically significant improvement (SMD = 0.32; 95% CI 0.05 to 0.59), with no observed heterogeneity. Although opioid outcomes could not be included in the meta-analysis due to reporting variability, narrative synthesis of three studies suggested consistent opioid-sparing effects. VR-related adverse events were rare, and dropout rates were minimal, reinforcing its safety and feasibility in ENT perioperative care.

These findings support virtual reality as a feasible and well-tolerated adjunct to perioperative ENT care, particularly for improving pain and patient satisfaction. Anxiety outcomes showed a non-significant trend toward benefit, and opioid reducing effects appear promising but remain inconclusive. Larger, standardized trials with longer follow-up are needed to confirm VR’s effectiveness in this setting.

## Introduction and background

Virtual reality (VR) technology has rapidly transitioned from entertainment and gaming into clinical medicine, where it is now being explored as a potential tool to improve pain management in different surgical and medical settings [1]. The use of VR in perioperative care creates an immersive environment that focuses on multisensory manipulation aiming to improve patients’ anxiety and level of discomfort following medical or surgical procedures. VR works by simulating alternate realities, in some cases set according to the patient’s preference, to divert their attention and modulate their perception, making it a unique tool to reduce recovery time and the need for pain control medications [1]. In recent years, VR has been trialled across surgical specialties as a non-pharmacologic adjunct to manage pain, alleviate anxiety, and reduce sedative and opioid requirements [2].

Within otorhinolaryngology and head and neck surgery, patients often undergo procedures associated with significant physical and psychological stress. These include both invasive operations such as sinus or airway surgery, and non-invasive office-based procedures like endoscopy or otomicroscopy. Regardless of the type of procedure, it has been shown that patients experience significant levels of stress and anxiety, resulting in high levels of pain and discomfort during and after the procedure [3]. Pain and anxiety in the perioperative period are distressing and tend to have a strong association with increased opioid consumption, longer recovery, and poorer patient satisfaction [3,4].

The theoretical basis for VR’s clinical utility lies in its capacity to activate attentional and emotional pathways [5]. By creating immersive, distracting environments, VR is thought to inhibit the brain’s processing of nociceptive stimuli through mechanisms consistent with the gate control theory of pain [5]. In studies investigating the neuroimaging of patients using VR, it was shown that VR modulates brain activities in cortical pain networks, including reduction of activation of the somatosensory cortex, insula, and anterior cingulate cortex [6]. In pediatric populations, VR has been particularly effective in mitigating procedural distress, with documented benefits during anesthesia induction, dental work, and venipuncture [7,8].

In addition to its impact on physical symptoms, VR has shown potential in enhancing procedural satisfaction and patient experience. Recent studies report improved patient engagement, reduced physiological stress, and greater perceived control during procedures involving minimal sedation [9,10]. These findings are particularly relevant to Ear, Nose and Throat (ENT) settings where patient cooperation and comfort are integral to procedural success.

Despite this emerging body of evidence, there remains a need to systematically evaluate the efficacy of VR interventions in ENT-related procedures. No prior meta-analysis or systematic review has focused exclusively on this surgical domain. Therefore, the objective of this review is to assess whether VR interventions, compared to standard care or control conditions, improve perioperative outcomes in ENT and head and neck procedures. Specifically, we evaluate their impact on postoperative pain, anxiety scores, opioid consumption, and patient satisfaction.

## Review

Methodology

Protocol and Registration

This systematic review and meta-analysis adhered to the Preferred Reporting Items for Systematic reviews and Meta-Analyses (PRISMA) 2020 guidelines and was registered with the International Prospective Register of Systematic Reviews (PROSPERO); registration ID: (CRD420251040287). The protocol details are publicly accessible.

Search Strategy

A comprehensive literature search of PubMed, EMBASE, and Cochrane CENTRAL databases was conducted from inception to April 2025. The search strategy used combinations of MeSH terms and keywords: ("virtual reality" OR "VR") AND ("head and neck surgery" OR "ENT surgery") AND (“pain” OR “anxiety” OR “opioid”). Both randomized and non-randomized study types that investigated VR use in the perioperative setting of ENT procedures were included. Included studies needed to have a control group (standard care) to compare the VR intervention to. There were no limits with regards to date of publication of the studies. Articles in languages other than English were excluded.

Study Selection and Data Extraction

Two reviewers (AA, MI) independently screened all titles and abstracts, followed by full-text review. All duplicates were removed using Rayyan software (https://www.rayyan.ai/) and cross-checked by two reviewers (AA, MI). Any uncertainty when deciding to include a study was resolved with collaboration with the remaining authors. Studies meeting all eligibility criteria had data extracted independently by two reviewers (AA, MI). The data extracted included information on study design, sample size, participant demographics, type and timing of surgery, characteristics of the virtual reality intervention, comparator group, and all reported outcomes. Primary outcomes included measures of postoperative pain, anxiety, opioid use and patient satisfaction. Secondary outcomes included comfort, procedural tolerability, and adverse events.

Risk of Bias Assessment

Risk of bias was accounted for by using the revised Cochrane Risk of Bias (RoB 2.0) tool [11] for randomized controlled trials (RCTs) and ROBINS-I tool [12] for the non-randomized controlled trial. Domains evaluated included the randomization process, deviations from intended interventions, missing outcome data, measurement of the outcome, and selection of the reported result. Randomized controlled trials were rated as low risk, some concerns, or high risk by RoB 2.0, while non-randomized control trials were rated as low, moderate, or serious risk by ROBINS-I.

Certainty of Evidence Assessment

The certainty of evidence for each outcome was assessed using the GRADE framework, considering factors such as risk of bias, inconsistency, indirectness, imprecision, and publication bias. A summary of these assessments is provided in the Appendix.

Statistical Analysis

Meta-analyses were conducted using standardized mean differences (SMDs), applying Hedges’ g to account for differences in measurement scales and small sample sizes. A random-effects model was used throughout, with heterogeneity quantified using the I² statistic. Subgroup analyses were performed based on participants’ age (adult vs. pediatric). Sensitivity analyses were conducted by excluding studies with higher risk of bias. Funnel plots were used to visually assess potential publication bias. All quantitative synthesis was performed using the ‘meta’ package in R (version 4.3.1; R Foundation for Statistical Computing, Vienna, Austria).

Results

Study Selection and Study Characteristics

**Figure 1:**
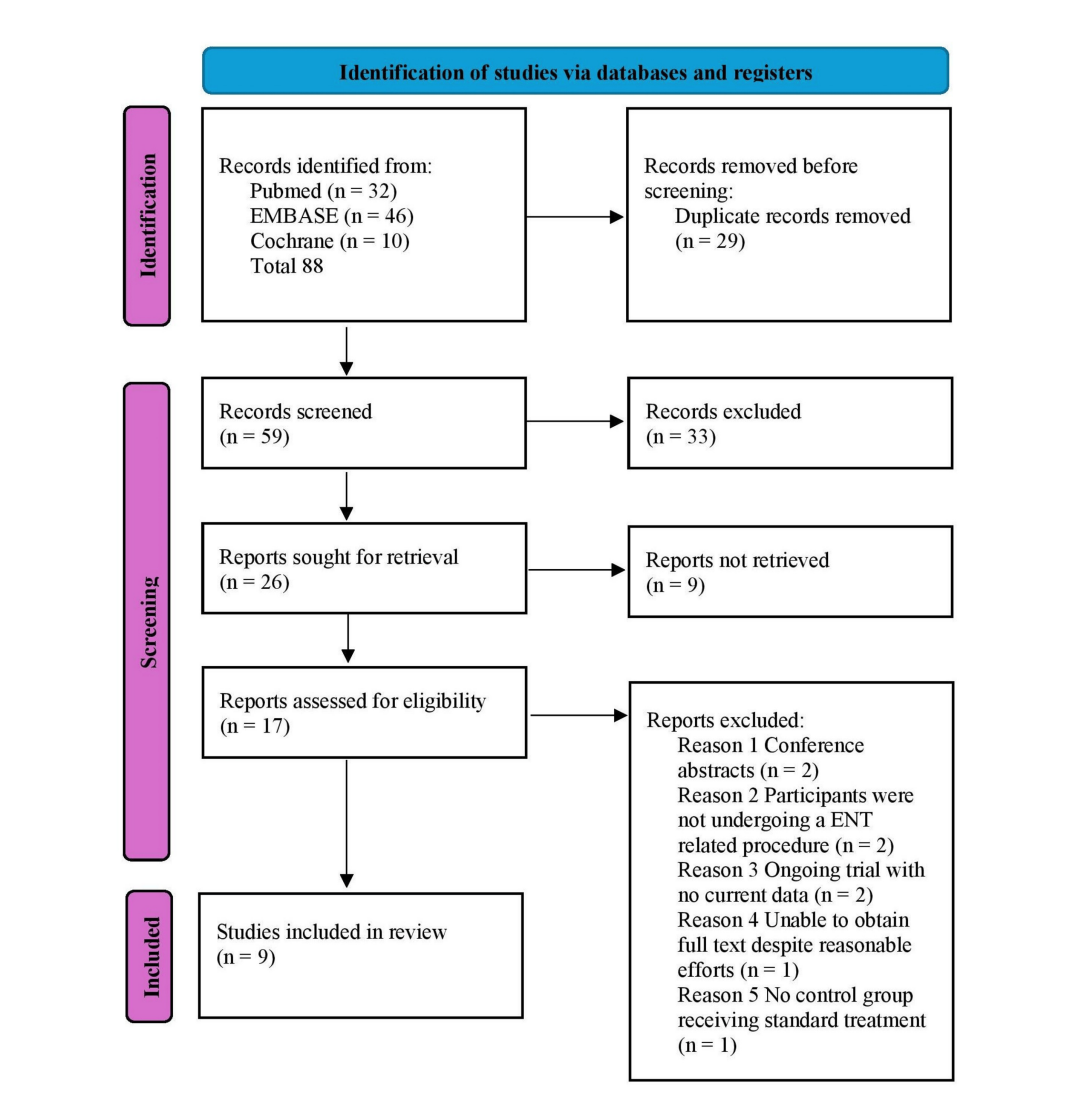
Preferred Reporting Items for Systematic reviews and Meta-Analyses (PRISMA) flow diagram illustrating the study selection process for inclusion in the systematic review and meta-analysis. A total of 88 records were identified, and nine studies that met the eligibility criteria were included in the final analysis.

A total of nine studies were included in this systematic review, following a comprehensive search of PubMed, EMBASE, and Cochrane databases. After removal of duplicates (n = 29), 59 records were screened, and 17 full-text reports were assessed for eligibility. Eight were excluded due to reasons including conference abstract format, non-ENT surgical populations, ongoing trials without data, or lack of available control groups, resulting in nine studies included in the final synthesis. The study selection process is demonstrated in the PRISMA flow diagram in Figure 1.

Among the included studies, eight were randomized controlled trials and one was a non-randomized controlled trial. These studies examined VR interventions across a variety of ENT and head and neck procedures, including otomicroscopy, nasal endoscopy, sinonasal endoscopy, laryngeal/tracheal procedures, head and neck surgery, esophageal manometry, ENT/dental procedures under general anesthesia, and outpatient laser procedures [13-21]. The total sample included 527 participants, ranging from pediatric to adult populations [13-21]. Three studies focused on pediatric patients [15,20,21], while the remaining six were conducted in adult populations [13,14,16-19]. A summary of these characteristics is seen in Table 1. 

**Table 1:** Summary of characteristics of the included studies Summary of key characteristics of the nine studies included in the systematic review and meta-analysis, detailing study design, participant demographics, surgical procedures, virtual reality interventions, and control conditions. Abbreviations: RCT: Randomized Controlled Trial; VR: Virtual Reality; GA: General Anesthesia; KTP laser: Potassium Titanyl Phosphate laser; 2D: Two-dimensional

Study	Design	Number of participants (VR Group)	Number of participants (Control group)	Population	Procedure	VR Type	VR Content	Control Group Intervention
Pandrangi et al. 2025 [13]	RCT (4-arm)	20	20	Adults	Inpatient ENT surgery	Oculus Quest	Mindfulness + Games	Standard postoperative care with no VR or wearable technology
Palte et al. 2024 [14]	RCT (2-arm)	20	20	Adults	Esophageal manometry	Oculus Quest	Mindfulness	Standard care with nasal anaesthesia (2% viscous lidocaine); no VR
Galst et al. 2024 [15]	RCT (3-arm)	20	19	Pediatric (<18)	Otomicroscopy	Pico G2 4K	Interactive game	Standard otomicroscopy without any distraction (no VR or tablet)
Zhao et al. 2023 [16]	Controlled Trial	23	14	Adults	KTP laser	Smartphone VR	Nature video	Standard in-office KTP laser procedure with topical anaesthesia; no audiovisual distraction
Pandrangi et al. 2022 [17]	RCT (2-arm)	14	15	Adults	Head and neck surgery	Oculus Quest	Angry Birds VR	Handheld 2D version of Angry Birds game on smartphone (non-immersive control)
Gray et al. 2021 [18]	RCT (Crossover)	43	39	Adults	Sinonasal endoscopy	Oculus Go	SpaceBurgers	Standard topical laryngotracheal and nasal anaesthesia; no distraction or VR
Chang et al. 2020 [19]	RCT (2-arm)	8	8	Adults	Laryngeal/tracheal procedures	Samsung Gear VR	Beach scene	Standard care using nasal phenylephrine and lidocaine spray; no VR
Liu et al. 2020 [20]	RCT (2-arm)	30	23	Pediatric (<18)	Nasal endoscopy	Oculus Go	SpaceBurgers	Topical nasal anaesthesia (lidocaine and Neo-Synephrine); no VR or distraction
Eijlers et al. 2019 [21]	RCT (Single-blind)	94	97	Pediatric (<18)	ENT/dental under GA	HTC Vive	Hospital simulation	Care-as-usual: online pre-op video + standard perioperative protocol; no VR

VR interventions were administered during different perioperative phases, preoperatively, intraoperatively, or postoperatively, using commercial headsets such as the Oculus Quest (Reality Labs, Menlo Park, CA, USA), Oculus Go (Reality Labs), Samsung Gear VR (Samsung Electronics, Suwon, South Korea), Pico G2 4K (ByteDance, Beijing, China), and HTC Vive (HTC Corporation, Taoyuan City, Taiwan) [13-21]. The VR content varied across studies and included passive experiences (e.g., nature environments, mindfulness exercises) as well as interactive modules (e.g., games, hospital simulations) [13-21]. The control groups generally received standard care without immersive distraction, with some studies incorporating non-immersive controls such as videos or handheld games [13-21].

**Figure 2:**
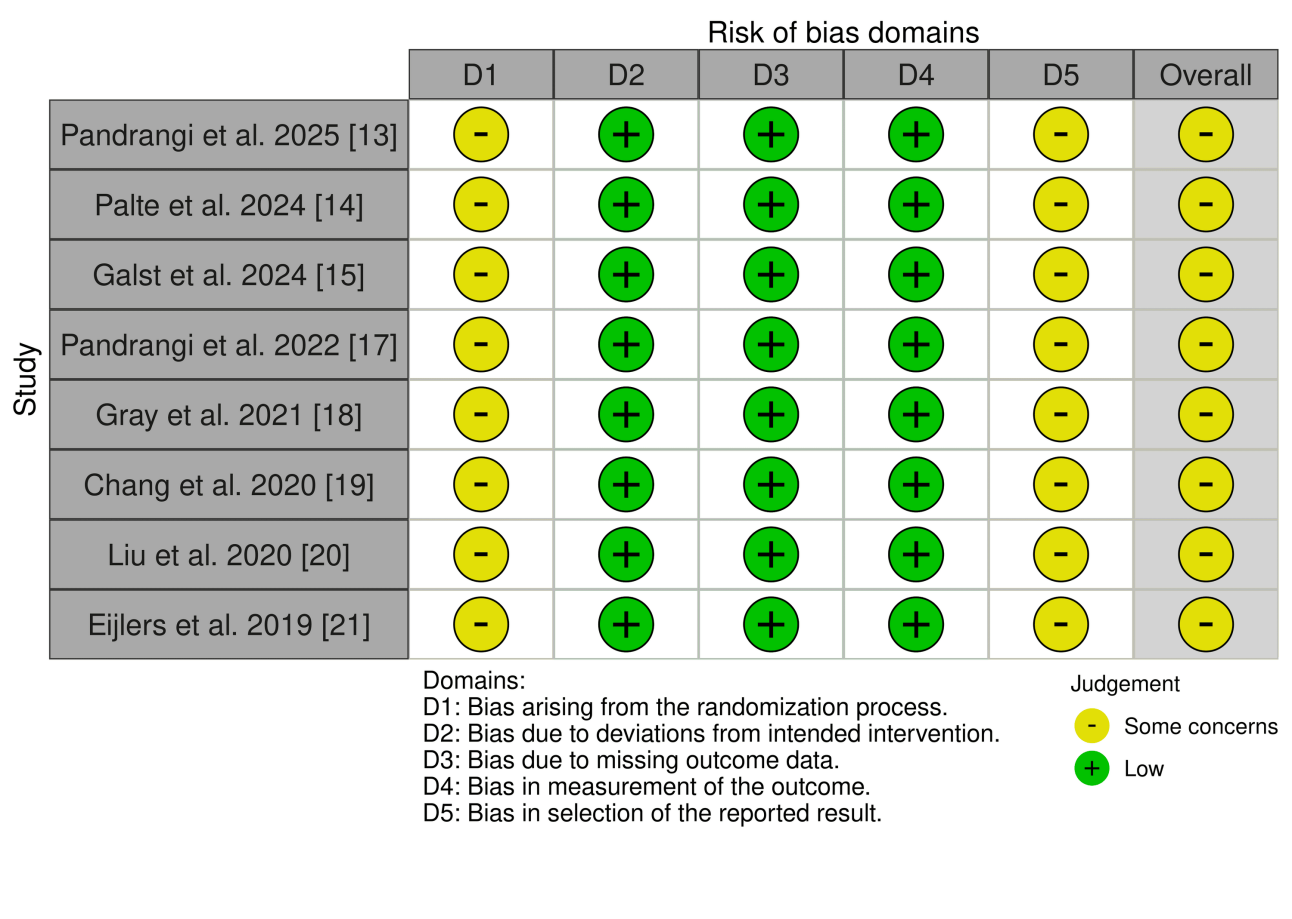
RoB 2 Tool Assessing Risk of Bias in Randomized Controlled Trial (RCT) Studies Summary of risk of bias assessments for randomized controlled trials included in the review, evaluated using the Cochrane RoB 2.0 tool. The figure was generated using the online visualization platform, robvis.

Risk of bias assessment using the RoB 2 tool indicated that all eight RCTs were rated as having "some concerns," primarily due to issues related to blinding, allocation concealment, or subjective outcome reporting (Figure 2). The one non-randomized trial was evaluated with the ROBINS-I tool and rated as having "moderate" risk of bias, mainly due to potential confounding and non-blinded outcome assessment​ (Table 2).

**Table 2:** ROBINS-I tool assessing risk of bias for non-randomized trial Risk of bias assessment for the non-randomized study included in the review, conducted using the ROBINS-I (Risk Of Bias In Non-randomized Studies - of Interventions) tool.

Study	Confounding	Selection Bias	Classification of Interventions	Deviations from Intended Interventions	Missing Data	Measurement of Outcomes	Selection of Reported Results	Overall
Zhao et al. 2023 [16]	Moderate	Low	Low	Low	Low	Moderate	Low	Moderate

Effect of Virtual Reality on Postoperative Pain Scores

A random-effects meta-analysis of five RCTs [15,17-20] and one non-RCT [16] was conducted to assess the efficacy of virtual reality interventions in reducing acute postoperative pain in patients undergoing ENT-related surgical procedures. These studies collectively included 256 participants (138 in VR groups and 118 in control groups), with pain outcomes measured immediately after the intervention. Pain was assessed using validated scales such as the Visual Analog Scale (VAS), Numeric Rating Scale (NRS), and Wong-Baker Faces Scale. Due to variability in the pain measurement instruments, the standardized mean difference was used to enable cross-study synthesis.

**Figure 3:**
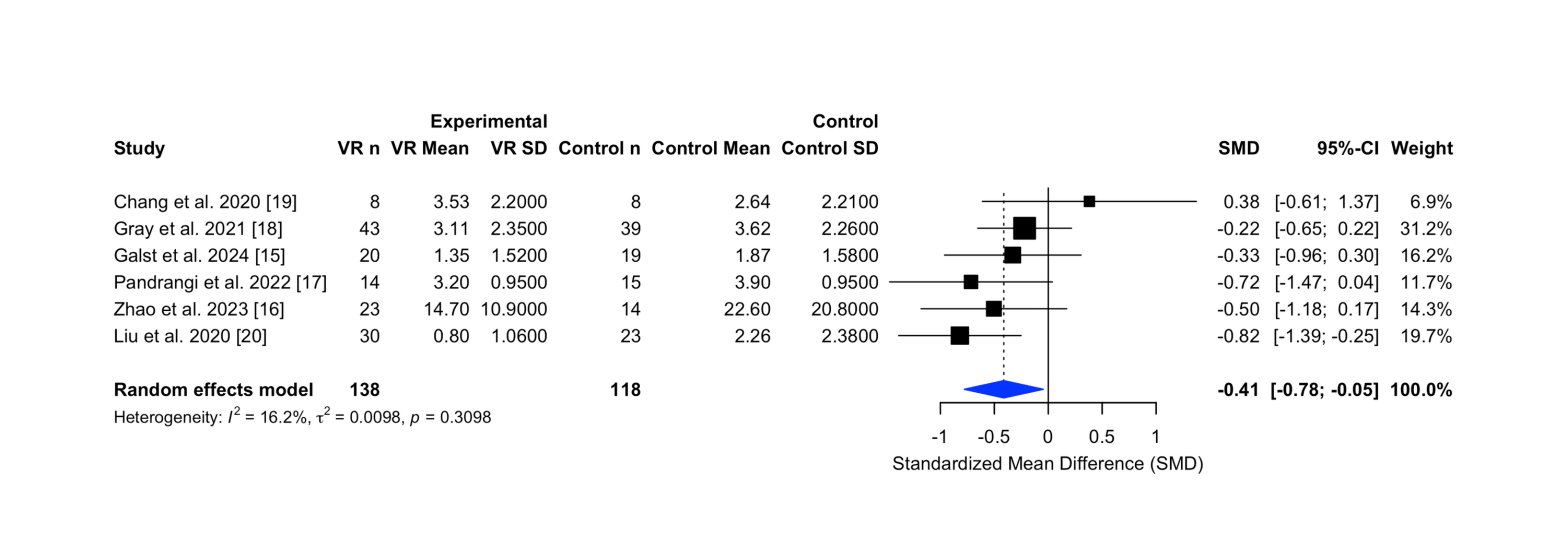
Forest plot comparing virtual reality versus control management for postoperative pain scores (random-effects meta-analysis). Forest plot comparing virtual reality interventions versus standard care for postoperative pain scores in Ear, Nose and Throat (ENT) surgical patients, based on a random-effects meta-analysis. The analysis demonstrated a moderate, statistically significant reduction in pain favoring virtual reality (VR). Figure generated using the R meta package. Abbreviations: VR n: number of participants in the VR group; VR Mean/SD: mean and standard deviation of the VR group; Control n: number of participants in the control group; Control Mean/SD: mean and standard deviation of the control group; SMD: Standardized Mean Difference; CI: Confidence Interval; I²: Higgins' I-squared; τ²: Tau-squared; p: p-value

The pooled effect estimate demonstrated a statistically significant reduction in postoperative pain scores, favoring VR over control conditions (SMD = -0.41, 95% CI -0.78 to -0.05). This effect represents a moderate treatment benefit. There was low heterogeneity across studies (I² = 16.2%, p = 0.3098), suggesting a consistent direction of effect across diverse clinical and demographic settings. These results are visualized in the forest plot in Figure 3.

Individual study estimates varied, with the strongest effect observed in Liu et al. (2020), a pediatric study reporting a large reduction in pain with VR [20]. Smaller or non-significant effects were observed in other studies, such as Chang et al. (2020) and Zhao et al. (2023), potentially due to small sample sizes or baseline differences [19,16]. Gray et al. (2021) contributed the most weight to the analysis due to its large sample size and precision [18].

Subgroup Analysis for Postoperative Pain Score

**Figure 4:**
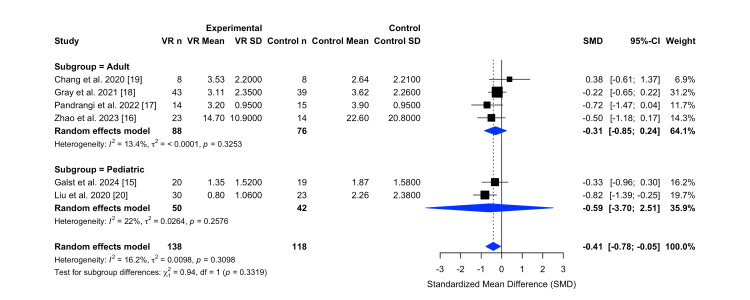
Subgroup analysis comparing virtual reality versus control interventions for postoperative pain in adult and pediatric populations. Subgroup forest plot comparing the effect of virtual reality interventions versus control on postoperative pain, stratified by adult and pediatric populations. The analysis explores differential treatment effects by age group and was generated using the R meta package. Abbreviations: VR n: number of participants in the VR group; VR Mean/SD: mean and standard deviation of the VR group; Control n: number of participants in the control group; Control Mean/SD: mean and standard deviation of the control group; SMD: Standardized mean difference; CI: Confidence interval; I²: I-squared; τ²: Tau-squared; χ²: Chi-squared test statistic; df: Degrees of freedom; p: p-value

A subgroup meta-analysis was conducted to examine whether the effectiveness of virtual reality interventions in reducing postoperative pain differed between adult and pediatric populations. Of the six included trials, four were categorized as adult-focused (n = 164; 88 in VR group, 76 in control group), and two involved pediatric populations (n = 92; 50 in VR group, 42 in control group). The analysis used standardized mean difference as the effect size to enable comparison across diverse pain scoring instruments. This subgroup analysis is seen in the Figure 4 forest plot. 

In adult trials, the pooled standardized mean difference was −0.31 (95% CI: −0.85 to 0.24), indicating a small to moderate reduction in postoperative pain favoring VR. However, the confidence interval crossed zero, suggesting that the effect was not statistically significant. Heterogeneity within this subgroup was low (I² = 13.4%, p = 0.3253), reflecting a consistent direction of treatment effect across studies [16-19].

In the pediatric subgroup, the pooled standardized mean difference was −0.59 (95% CI: −3.70 to 2.51), indicating a reduction in postoperative pain favoring VR. However, the wide confidence interval suggests a lack of statistical significance, likely influenced by the small sample size. Heterogeneity within pediatric studies was low (I² = 22%, p = 0.2576), indicating consistent direction of effect across trials [15,20].

The test for subgroup differences (χ² = 0.94, df = 1, p = 0.3319) indicated that there was no statistically significant difference in the effect of VR on postoperative pain between adult and pediatric populations. This suggests that the observed variation in effect sizes across subgroups is likely due to chance rather than a true difference in treatment efficacy by age group.

Sensitivity Analysis for Postoperative Pain Scores

**Figure 5:**
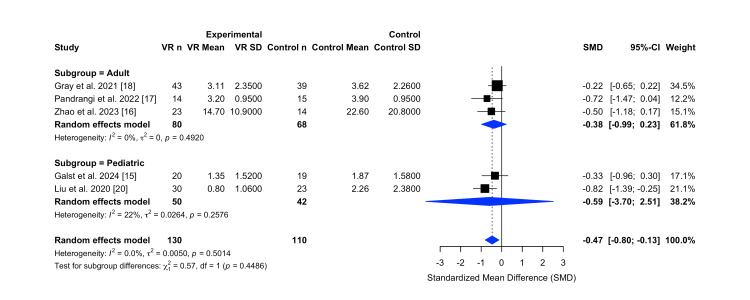
Sensitivity analysis excluding Chang et al. (2020): forest plot comparing virtual reality versus control for postoperative pain. Sensitivity analysis forest plot evaluating the effect of virtual reality on postoperative pain following exclusion of Chang et al. (2020) [19], which demonstrated a paradoxical direction of effect. The analysis was conducted using a random-effects model in the R meta package. Abbreviations: VR n: number of participants in the VR group; VR Mean/SD: mean and standard deviation of the VR group; Control n: number of participants in the control group; Control Mean/SD: mean and standard deviation of the control group; SD: Standard deviation; SMD: Standardized mean difference; CI: Confidence interval; I²: I-squared; τ²: Tau-squared; χ²: Chi-squared test statistic; df: Degrees of freedom; p: p-value

A sensitivity analysis was conducted after excluding Chang et al. (2020) due to its paradoxical direction of effect [19]. The final analysis included five studies, comprising 130 participants in the VR group and 110 in the control group [15-18,20]. This analysis is visualized in Figure 5.

The pooled standardized mean difference was -0.47 (95% CI: -0.80 to -0.13) under a random-effects model, indicating a statistically significant and moderate reduction in postoperative pain favoring VR interventions. Heterogeneity was negligible (I² = 0.0%, τ² = 0.0050, p = 0.5014), indicating strong consistency across studies.

In the adult subgroup, the pooled SMD was -0.38 (95% CI: -0.99 to 0.23), suggesting a small to moderate reduction in pain, though this was not statistically significant. Heterogeneity remained absent (I² = 0%, p = 0.4920). In contrast, the pediatric subgroup showed a larger effect size (SMD = -0.59; 95% CI: -3.70 to 2.51), though with a wide confidence interval due to limited sample size.

The test for subgroup differences (χ² = 0.57, df = 1, p = 0.4486) indicated no statistically significant difference between adult and pediatric populations, reinforcing that both age groups may derive comparable benefits from VR interventions.

**Figure 6:**
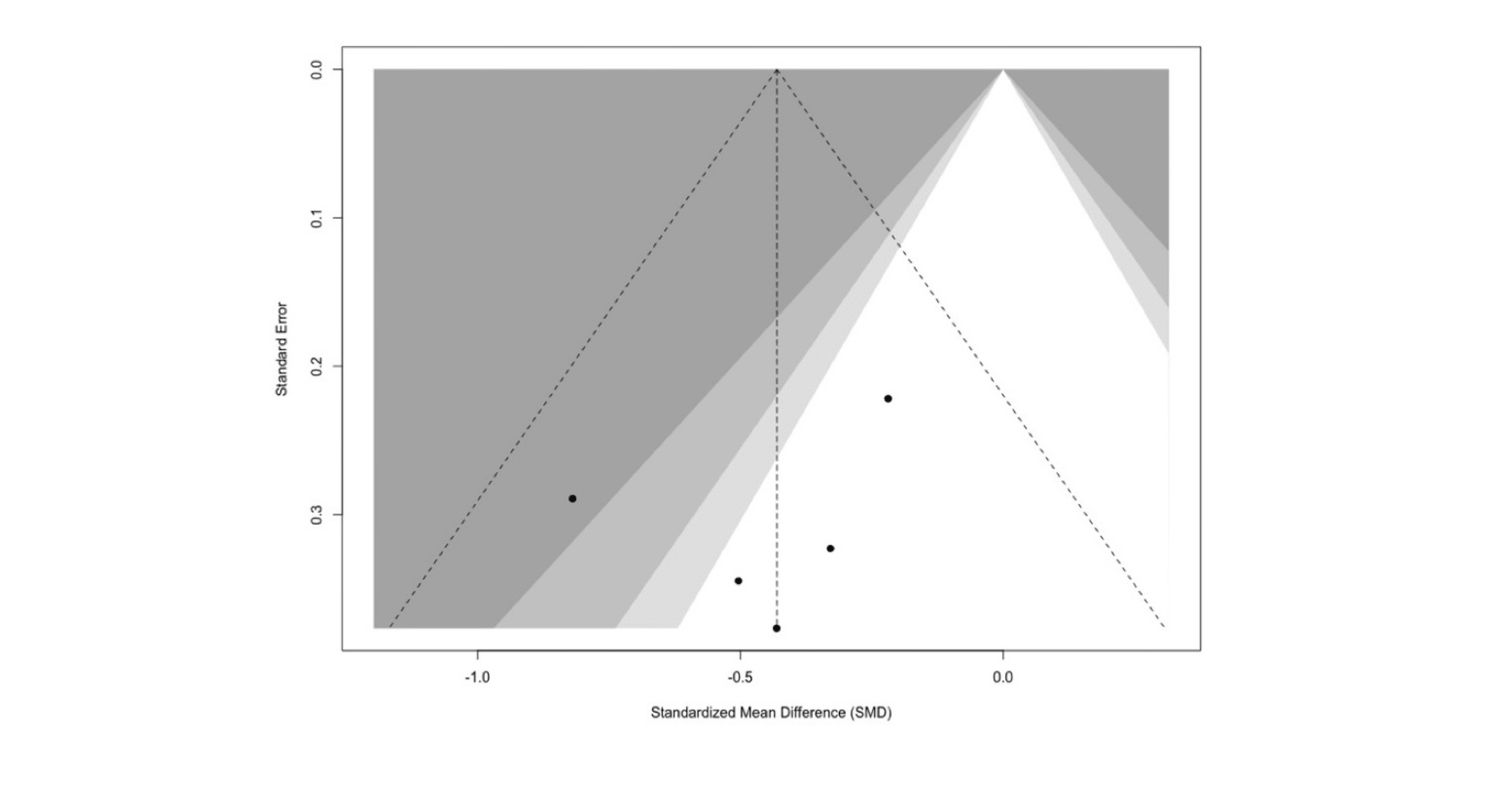
Funnel plot assessing publication bias for postoperative pain outcome following sensitivity analysis. Funnel plot assessing publication bias for postoperative pain outcomes based on the sensitivity analysis. The symmetrical distribution suggests a low likelihood of small-study effects [15-18,20]. Figure generated using the R meta package.

The funnel plot (Figure 6) of the five included studies appears symmetrical around the pooled standardized mean difference, suggesting a low risk of publication bias. Study points are distributed relatively evenly on both sides of the vertical line, and none fall far outside the pseudo 95% confidence contours.

Effect of Virtual Reality on Peri-operative Anxiety

Three studies were included in the meta-analysis evaluating the effect of VR on procedural anxiety in ENT settings, comprising a total of 172 participants (96 in the VR group and 76 in the control group) [16,18,20]. The pooled SMD was -0.85 (95% CI: -1.88 to 0.18), suggesting a moderate reduction in anxiety favoring VR, though this did not reach statistical significance.

**Figure 7:**
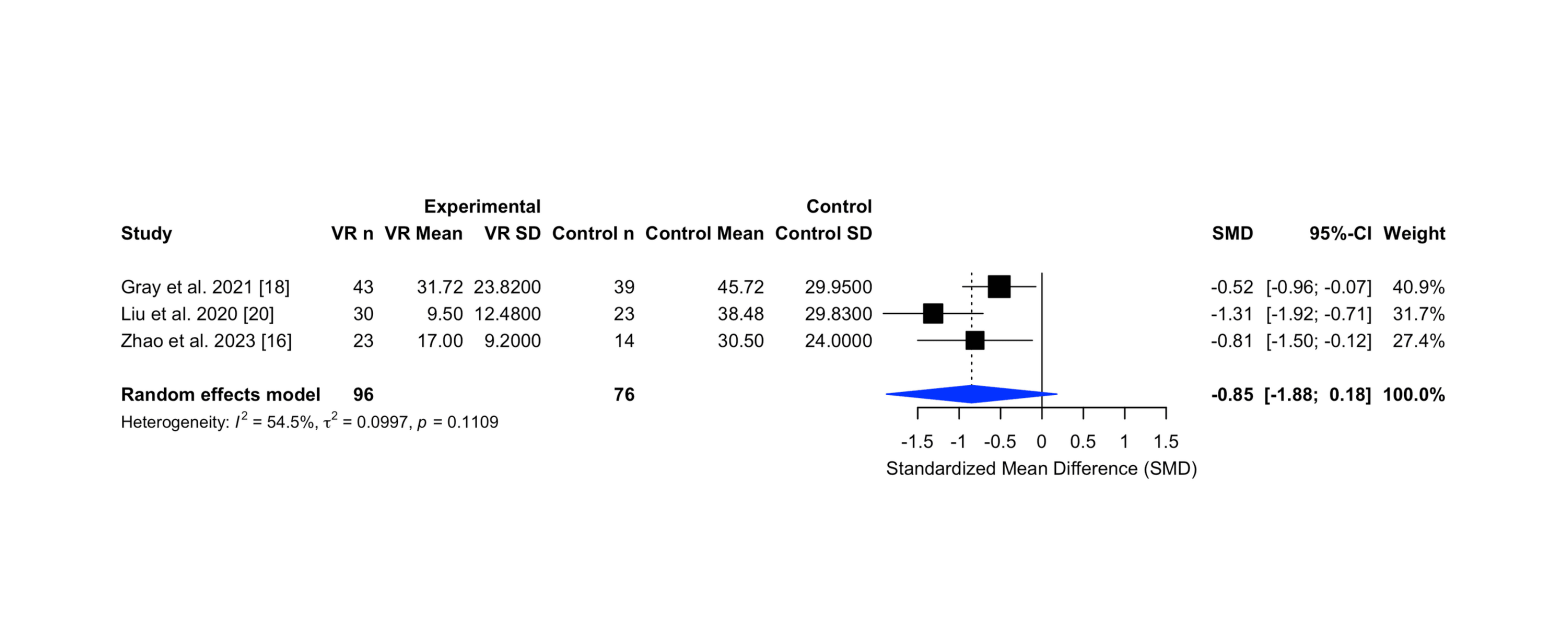
Forest plot comparing virtual reality versus control interventions in perioperative anxiety scores: random-effects meta-analysis. Forest plot comparing the effects of virtual reality versus control interventions on perioperative anxiety in Ear, Nose and Throat (ENT) surgical patients, using a random-effects model. The analysis showed a statistically significant reduction in anxiety favoring virtual reality (VR). Plot generated using the R meta package. Abbreviations: VR n: number of participants in the VR group; VR Mean/SD: mean and standard deviation of the VR group; Control n: number of participants in the control group; Control Mean/SD: mean and standard deviation of the control group; SD: Standard deviation; SMD: Standardized mean difference; CI: Confidence interval; I²: I-squared; τ²: Tau-squared; p: p-value

Heterogeneity was moderate (I² = 54.5%, τ² = 0.0997, p = 0.1109), indicating some variability in study designs or populations, yet with a consistent direction of effect. These results are presented in the Figure 7 forest plot.

**Figure 8:**
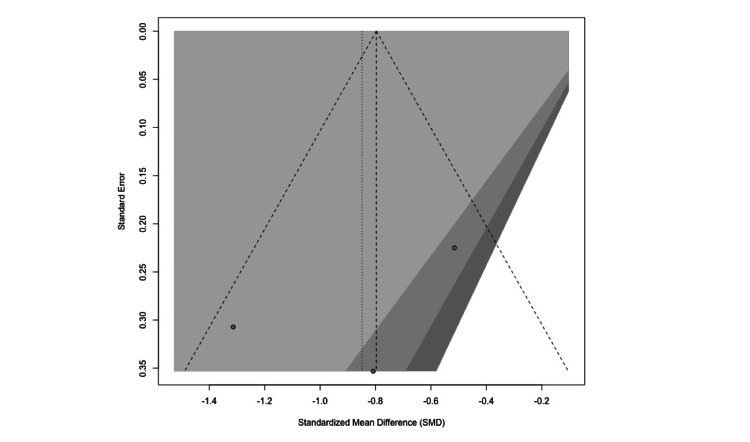
Funnel plot assessing publication bias for perioperative anxiety outcomes. Funnel plot assessing publication bias for perioperative anxiety outcomes. The visual distribution suggests minimal risk of small-study effects [16,18,20]. Figure generated using the R meta package.

Visual inspection of the funnel plot indicated a generally symmetrical distribution of studies, suggesting a low risk of publication bias (Figure 8).

Anxiety was assessed using validated instruments across all three studies. Gray et al. (2021) and Liu et al. (2020) utilized the Subjective Units of Distress Scale (SUDS), a 0-100 self-report scale for perceived anxiety [18,20]. Gray et al. (2021) reported that adult patients undergoing nasal endoscopy showed significantly lower anxiety scores in the VR group compared to control (31.7 ± 23.8 vs. 45.7 ± 29.9; p = 0.043) [18]. Liu et al. (2020) demonstrated substantial reductions in pediatric patients’ anxiety scores (9.5 ± 12.5 vs. 38.5 ± 29.8; p = 0.0002), along with significant reductions in caregiver anxiety [20]. Zhao et al. (2023) employed the State-Trait Anxiety Inventory (STAI-S) and a 100-mm visual analogue scale, also finding significantly lower stress scores in the VR group (17.0 ± 9.2) compared to control (30.5 ± 24.0; p = 0.021) [16].

Effects of Virtual Reality on Postoperative Opioid Use

Only three of the nine included studies reported outcomes related to opioid use, and due to inconsistent reporting formats and missing quantitative group-level data, a meta-analysis could not be performed. Nevertheless, these studies provide preliminary insights into the potential role of VR in reducing perioperative opioid consumption.

Pandrangi et al. (2025) evaluated a multimodal digital recovery intervention involving VR and wearable activity trackers in patients undergoing head and neck surgery [13]. Patients in the combined VR + Fitbit group demonstrated significantly lower average daily opioid use compared to controls (8.8 ± 20.6 morphine milligram equivalents (MME) vs. 26.4 ± 37.4 MME; p = 0.02) [13]. Notably, this significant reduction was not observed in the Fitbit-only group, suggesting that the opioid-sparing effect was driven by the combined use of VR and activity tracking rather than the wearable tracker alone. Additionally, opioid consumption on days with device engagement (either VR use or ≥2,000 steps) was significantly reduced relative to non-engaged days (15.0 ± 35.6 MME vs. 30.0 ± 52.5 MME; p = 0.004) [13].

Pandrangi et al. (2022) found that VR was associated with significant reductions in postoperative opioid use after head and neck surgery [17]. Compared to baseline, opioid consumption decreased by 9.1 MME (95% CI: -15.0 to -1.3 MME; d = 0.90) within four hours, and by 14.0 MME (95% CI: -25.6 to -2.4 MME; d = 0.94) within eight hours of VR intervention [17].

Eijlers et al. (2019) focused on pediatric patients undergoing adenoidectomy and tonsillectomy under general anesthesia, which involved either intravenous induction with propofol and fentanyl or inhalational induction with sevoflurane, followed by maintenance with sevoflurane in oxygen/air mixture and routine perioperative analgesia including paracetamol and diclofenac [21]. Although pain scores were not significantly different, the proportion of children requiring rescue analgesia (morphine) postoperatively was significantly lower in the VR group than in controls (55.0% vs. 95.7%; p = 0.002), suggesting an indirect opioid-sparing effect [21].

Patient Satisfaction Outcomes Following Virtual Reality Interventions

Five studies were included in the meta-analysis evaluating patient satisfaction following the use of virtual reality in ENT procedures [13,15,16,18,19]. Satisfaction scores were reported using a variety of scales, including Likert-type scales, 100-point visual analog scales, and hospital-specific instruments [13,15,16,18,19]. To account for these differences, standardized mean differences were calculated using a random-effects model.

**Figure 9:**
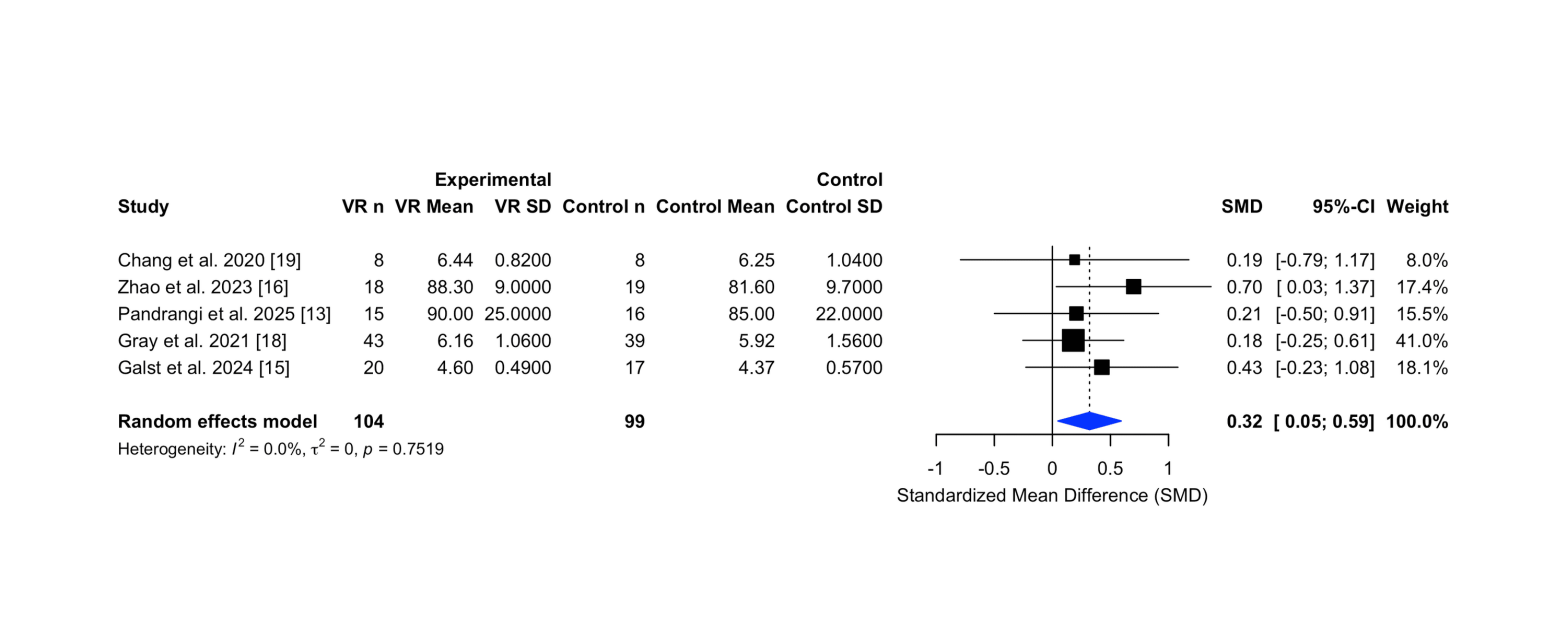
Forest plot illustrating the effect of virtual reality interventions on patient satisfaction scores compared to control groups in ENT procedures. Forest plot illustrating the effect of virtual reality (VR) interventions on patient satisfaction in Ear, Nose and Throat (ENT) surgical settings, compared to standard care. The random-effects meta-analysis demonstrated a small but statistically significant improvement in satisfaction scores favoring VR, with no observed heterogeneity across studies. Figure generated using the R meta package. Abbreviations: VR n: number of participants in the VR group; VR Mean/SD: mean and standard deviation of the VR group; Control n: number of participants in the control group; Control Mean/SD: mean and standard deviation of the control group; SD: Standard deviation; SMD: Standardized mean difference; CI: Confidence interval; I²: I-squared; τ²: Tau-squared; p: p-value

The pooled SMD was 0.32 (95% CI: 0.05 to 0.59), indicating a statistically significant improvement in satisfaction among participants receiving VR interventions compared to controls. Heterogeneity was low (I² = 0%, τ² = 0, p = 0.7519), suggesting consistency in effect estimates across studies despite the variability in measurement tools and settings. These results are visualized in the forest plot in Figure 9. 

**Figure 10:**
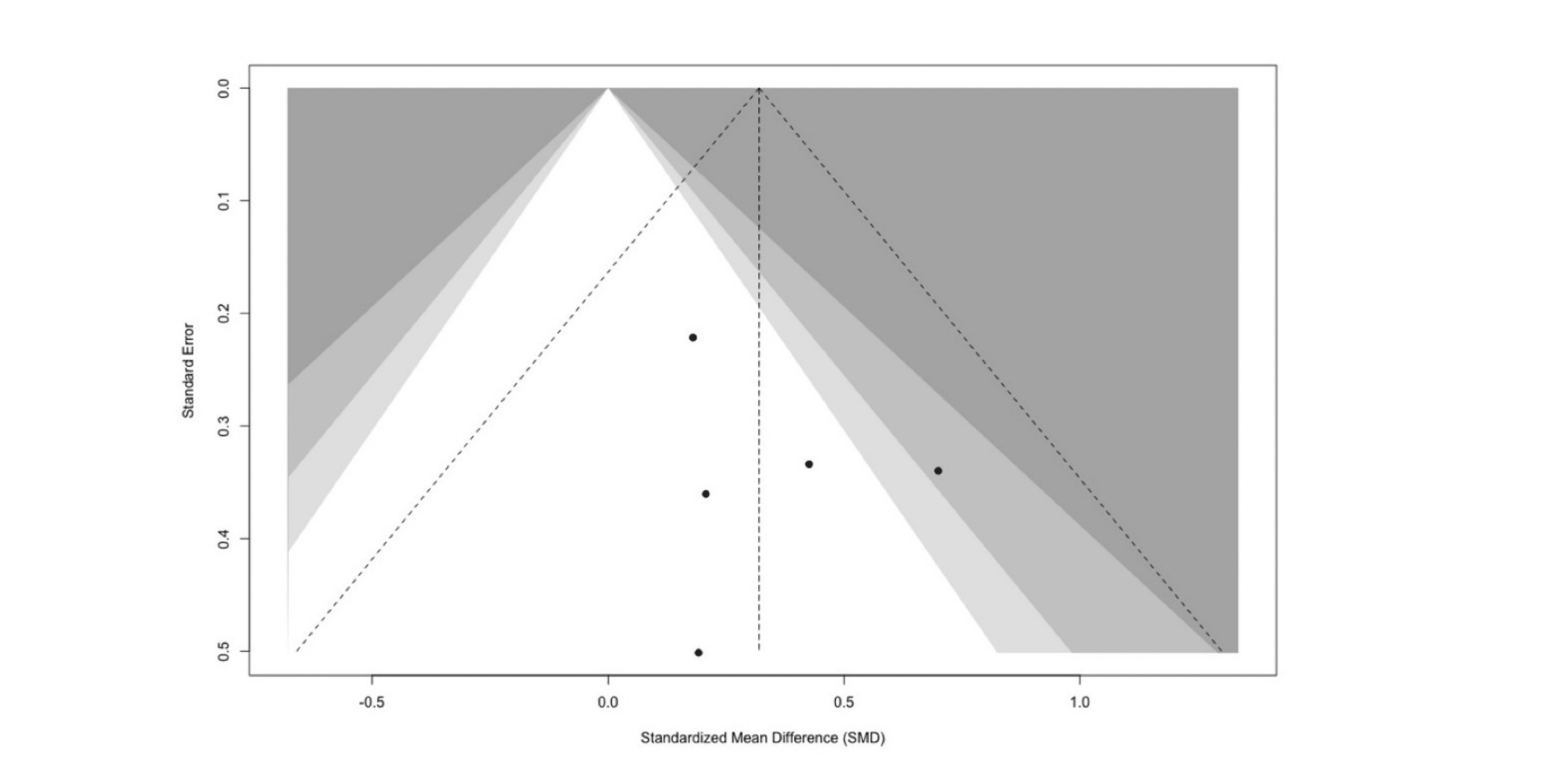
Funnel plot assessing publication bias for studies evaluating patient satisfaction outcomes following virtual reality interventions. Funnel plot assessing publication bias for studies evaluating patient satisfaction outcomes following virtual reality interventions in Ear, Nose and Throat (ENT) procedures. The symmetrical distribution of study estimates suggests low risk of small-study effects or reporting bias [13,15,16,18,19]. Figure generated using the R meta package.

The funnel plot (Figure 10) demonstrated a symmetrical distribution of studies around the effect estimate, with no indication of small-study effects or publication bias for the analysis of the patient satisfaction outcome. 

Other Outcomes

In addition to primary outcomes such as pain, anxiety, opioid use and patient satisfaction, several studies reported secondary experiential outcomes related to procedural comfort, VR tolerability, and clinician-perceived feasibility. These collectively provide insights into the broader acceptability and utility of virtual reality as a perioperative adjunct in ENT and head and neck settings.

Comfort and emotional experience were explored in a subset of trials. Palte et al. (2024) noted improved patient calmness as measured by the STAI-6 calmness subscale and a significantly slower galvanic skin response rise, reflecting reduced physiological stress during esophageal manometry [14]. Galst et al. (2024) found that children undergoing otomicroscopy rated the experience as significantly more positive in the VR group compared to control, despite no differences in pain scores [15]. In both cases, the immersive quality of VR was credited for improved procedural tolerance.

Procedural feasibility and device tolerability were explicitly addressed in several studies. Chang et al. (2020) and Gray et al. (2021) reported that VR did not interfere with clinician workflow, and patients maintained procedural compliance [19,18]. Gray et al. additionally measured reflexive head movements and found that VR significantly reduced spontaneous movements during sinonasal procedures, potentially contributing to improved procedural control and safety [18]. No studies reported interference with surgical equipment or disruption in standard care delivery [13-21].

Adverse effects and dropout rates were minimal across all studies [13-21]. None of the included trials reported VR-related side effects such as nausea, dizziness, disorientation, or headset-related discomfort [13-21]. These findings were consistent across pediatric and adult populations [13-21].

Patient and caregiver acceptance was particularly notable in pediatric settings. In Eijlers et al. (2019), children and parents expressed high satisfaction with the VR experience [21]. Similarly, Liu et al. (2020) found universal preference for VR among children, with additional caregiver-reported reductions in anxiety and procedural distress [20].

Discussion

This systematic review and meta-analysis evaluated the role of virtual reality as a perioperative adjunct in ENT surgery, demonstrating promising outcomes across several domains. Notably, VR interventions were associated with reductions in postoperative pain and anxiety, improvements in patient satisfaction, and potential reductions in opioid use. These findings highlight VR’s potential as a non-pharmacological, low-risk tool to enhance perioperative care in both pediatric and adult ENT populations.

The sensitivity meta-analysis for postoperative pain, which excluded the Chang et al. (2020) study due to its paradoxical direction of effect, revealed a statistically significant and moderate reduction in pain favoring VR (SMD = -0.47, 95% CI: -0.80 to -0.13). Heterogeneity was negligible (I² = 0%, p = 0.5014), suggesting a consistent treatment effect across the remaining five studies. These findings align with prior evidence demonstrating VR’s analgesic potential in other surgical populations, including orthopedic and burn patients [22,23]. For example, Morris et al. (2010) found that VR significantly reduced acute pain intensity during physical therapy for burns, reinforcing the concept that immersive distraction can meaningfully modulate pain perception [23]. These pain-reducing effects can be explained by studies that suggest VR attenuates nociceptive processing at the cortical level by engaging attentional and affective networks, diminishing activity in regions such as the anterior cingulate cortex and somatosensory cortices [23]. Mechanistically, VR’s ability to divert cognitive focus away from painful stimuli may explain the consistent pain reductions observed across our included studies, despite variability in surgical procedure and VR content [22].

Subgroup analysis by age indicated a larger effect size in pediatric populations compared to adults, although this difference did not reach statistical significance. This trend is consistent with the literature, where children have been shown to experience greater immersion and emotional engagement with VR environments, possibly amplifying the analgesic benefit [9]. Piskorz and Czub (2018) reported that pediatric patients undergoing venipuncture exhibited greater reductions in pain scores when using VR compared to standard distraction methods, suggesting that VR may exploit developmental differences in attentional capacity to enhance analgesia [10].

The meta-analysis evaluating anxiety outcomes demonstrated a moderate reduction in procedural anxiety favoring VR (SMD = -0.85, 95% CI: -1.88 to 0.18); however, this result did not reach statistical significance. Heterogeneity was moderate (I² = 54.5%, p = 0.1109), indicating some variation across study populations and designs.These results are particularly relevant considering the heightened prevalence of preoperative anxiety in ENT patients, which has been associated with poorer surgical outcomes, increased postoperative pain, and greater anesthesia requirements [24]. Although our findings did not reach statistical significance, they align with existing literature, including findings by Tanja-Dijkstra et al. (2018), who observed anxiety reductions with VR during dental procedures [25]. Additionally, neurobiological research suggests that VR may modulate limbic system activity, including the amygdala, a key region involved in the anxiety response, providing a potential neurophysiological basis for the anxiolytic effects observed [26]. These findings suggest that VR may be an effective and adaptable tool for anxiety management in ENT surgeries, with meaningful implications for improving patient experience and potentially influencing perioperative outcomes.

Despite promising findings for pain and anxiety, the evaluation of VR’s impact on opioid use was limited by heterogeneity in reporting formats, precluding quantitative synthesis. Nevertheless, the three studies consistently suggested that VR interventions may reduce postoperative opioid requirements, echoing results from broader surgical fields [13,17,21]. Spiegel et al. (2019), in a randomized controlled trial among hospitalized patients, demonstrated that VR was associated with decreased pain and lower opioid prescriptions, providing further support for VR’s role in addressing postoperative analgesic needs without pharmacologic escalation [4]. 

Patient satisfaction outcomes further indicate the clinical relevance of VR. The pooled analysis demonstrated a small but statistically significant improvement in satisfaction scores among participants receiving VR interventions compared to controls (SMD = 0.32, 95% CI 0.05 to 0.59), with no heterogeneity across studies. These findings are supported by Ayari et al. (2025), who reported that immersive VR significantly enhanced patient satisfaction during outpatient colonoscopy without sedation [27]. Given that satisfaction with care is increasingly recognized as a critical quality metric associated with better adherence, reduced perioperative anxiety, and improved recovery trajectories, even modest improvements driven by VR use could have meaningful clinical implications [27]. Thus, VR’s ability to enhance patient satisfaction may extend benefits beyond the immediate perioperative encounter, potentially contributing to broader quality-of-care outcomes.

Secondary outcomes across studies consistently highlighted the favorable acceptability, feasibility, and safety of VR interventions. No adverse events related to VR use were reported, and dropout rates were minimal, consistent with prior meta-analyses in non-ENT populations [28]. These findings are reassuring given theoretical concerns about VR-induced cybersickness or exacerbation of anxiety, especially in pediatric cohorts.

Limitations

This systematic review and meta-analysis had several limitations regarding the interpretation of our findings. The overall sample sizes were relatively small, which may have reduced the precision of pooled estimates and introduced the risk of inflated effect sizes due to small-study effects; however, funnel plots did not indicate substantial publication bias. Moreover, risk of bias assessments identified some concerns in most included studies, primarily related to the challenges of blinding participants and outcome assessors to VR interventions. Clinical heterogeneity, particularly in VR content (e.g., passive nature scenes versus interactive games), delivery timing (pre-, intra-, or postoperatively), and exposure duration, also warrants caution when generalizing these findings. Measurement heterogeneity across studies further complicated data synthesis, though the use of standardized mean differences helped mitigate this limitation.

Importantly, while pooled results for postoperative pain and patient satisfaction reached statistical significance, the anxiety outcome did not, and confidence intervals for subgroup analyses were wide. This stresses the need for larger, more adequately powered trials to confirm these effects and reduce uncertainty in subgroup comparisons.

Opioid outcomes suffered from inconsistent reporting and were limited to narrative synthesis, highlighting the need for standardized opioid-related endpoints in future VR research. Additionally, most studies assessed short-term outcomes; thus, the durability of VR’s benefits into the postoperative recovery period remains unknown. Finally, all included studies were conducted in single-center settings, potentially limiting external validity.

Despite these limitations, the consistency of findings across diverse procedures, patient demographics, and outcome measures suggests a meaningful clinical signal favoring VR intervention. The integration of VR into perioperative ENT care can potentially offer a non-invasive, scalable, and patient-centered strategy to address pain, anxiety and overall patient satisfaction. 

## Conclusions

In summary, virtual reality represents a promising adjunct to perioperative care in ENT surgery, offering statistically significant reductions in postoperative pain, improved patient satisfaction, and potential opioid-sparing effects. While the pooled results also suggest a reduction in perioperative anxiety, this did not reach statistical significance, highlighting the need for larger confirmatory studies. Our systematic review and meta-analysis support VR as a safe, feasible, and well-tolerated intervention, with no reported adverse events and minimal attrition. These findings highlight its potential to enhance the patient experience in the perioperative setting. However, heterogeneity in intervention protocols, small sample sizes, and inconsistent opioid reporting highlight the need for more rigorous trials. Future research should focus on standardizing outcome measures, evaluating long-term efficacy, and assessing cost-effectiveness in real-world settings. With continued refinement, VR could become an accessible, evidence-based tool to improve perioperative outcomes in ENT and broader surgical contexts.

## Appendices

**Table 3:** GRADE Certainty Assessment of Result Outcomes The certainty of evidence for each outcome was assessed using the GRADE framework and is summarized here. Overall, the evidence ranged from moderate to low certainty across outcomes. Postoperative pain and patient satisfaction outcomes were graded as moderate certainty, while anxiety and opioid use outcomes were downgraded to low certainty due to concerns related to inconsistency, imprecision, and reporting limitations. RCT: Randomized controlled trial

Outcome	Number of Studies	Study Design	Risk of Bias	Inconsistency	Indirectness	Imprecision	Publication Bias	Overall Certainty	Reference
Postoperative Pain	5 studies (sensitivity meta-analysis)	RCTs + 1 non-RCT	Some concerns	Low (I² = 0% after sensitivity analysis)	No serious indirectness	No serious imprecision	Low risk (symmetrical funnel plot)	Moderate	[15-18,20]
Anxiety	3 studies (meta-analysis)	RCTs	Some concerns	Moderate (I² = 54.5%)	No serious indirectness	Some imprecision (small sample sizes)	Low risk (symmetrical funnel plot)	Low to Moderate	[16,18,20]
Opioid Use	3 studies (narrative synthesis)	RCTs + 1 non-RCT	Some concerns	Not applicable (narrative synthesis)	Some indirectness (different opioid reporting formats)	Serious imprecision	Not assessable	Low	[13,17,21]
Patient Satisfaction	5 studies (meta-analysis)	RCTs + 1 non-RCT	Some concerns	Low (I² = 0%)	No serious indirectness	No serious imprecision	Low risk (symmetrical funnel plot)	Moderate	[13,15,16,18,19]
